# Second‐line treatment options in advanced thymic carcinoma after failure of platinum‐based chemotherapy: A multicenter retrospective study

**DOI:** 10.1002/cam4.5053

**Published:** 2022-08-04

**Authors:** Yang Wang, Xuanye Zhang, Dan Tian, Sen Han, Jie Zhang, Jun Nie, Ling Dai, Weiheng Hu, Xiaoling Chen, Xiangjuan Ma, Guangming Tian, Di Wu, Ziran Zhang, Jieran Long, Jian Fang

**Affiliations:** ^1^ Key Laboratory of Carcinogenesis and Translational Research (Ministry of Education/Beijing), Department of Thoracic Oncology Peking University Cancer Hospital & Institute Beijing China; ^2^ State Key Laboratory of Oncology in South China, Collaborative Innovation Center for Cancer Medicine, Department of Medical Oncology Sun Yat‐sen University Cancer Center Guangzhou China; ^3^ Department of Thoracic Surgery, Guangdong Provincial People's Hospital Guangdong Academy of Medical Sciences Guangzhou China

**Keywords:** docetaxel, PD‐1 inhibitor, platinum, second‐line therapy, thymic carcinoma

## Abstract

**Background:**

Currently there is no standard therapy recommended for second‐line treatment for thymic carcinoma. Our study compared multidrug chemotherapy, single‐agent chemotherapy, and PD‐1 inhibitors in patients diagnosed with advanced thymic carcinoma who had previous platinum‐based chemotherapy at the clinic.

**Methods:**

The study included patients with thymic carcinoma who failed first‐line platinum‐based chemotherapy. Kaplan–Meier methods were applied in the study for estimating the progression‐free survival (PFS) and overall survival (OS) curves. Pearson chi‐square or Fisher's exact chi‐square test was adopted to make comparisons of the objective response rate (ORR) between treatment groups. Cox regression was used for the multivariate analyses in PFS and OS.

**Results:**

Among the 92 patients enrolled, multidrug chemotherapy was used in 51 (55.4%) patients for second‐line therapy. Thirty‐six patients (35.9%) received single‐agent chemotherapy, and eight patients (8.7%) underwent PD‐1 inhibitors. The multidrug chemotherapy group showed better efficacy than the other two groups, with an ORR of 35.3% (*p* = 0.006). The median PFS of multidrug chemotherapy, single‐agent chemotherapy and PD‐1 inhibitors were 5.0 months, 3.0 months, and 4.0 months, respectively (*p* = 0.008). Patients in the multidrug chemotherapy group also showed an advantage in OS in comparison with the other two treatment groups (*p* = 0.045), with a median OS of 30.4 months. Multivariate analysis showed that second‐line treatment was independent factor for both PFS (*p* = 0.035) and OS (*p* = 0.037). Grade 3–4 AEs were mostly detected in patients receiving multidrug chemotherapy and were primarily hematologic. Treatment‐related mortality was not found in any of the groups.

**Conclusions:**

Multidrug chemotherapy had a trend toward a more positive response rate and outcomes in longer survival time than single‐agent chemotherapy and PD‐1 inhibitors. Multidrug chemotherapy is a choice worth considering for second‐line therapy in patients with thymic carcinoma if tolerable.

## Graphical abstract

(1) In 92 patients, the multidrug chemotherapy group showed better efficacy than the single‐agent chemotherapy group and the PD‐1 inhibitors group, with an ORR of 35.3%, 6.0%, and 12.5%, respectively (*p* = 0.006). (2) In second‐line therapy, the median PFS was 5.0 months for multidrug chemotherapy, 3.0 months for monotherapy and 4.0 months for PD‐1 inhibitors (*p* = 0.008). (3) The multidrug chemotherapy group had an advantage over the other groups in OS (*p* = 0.045), with median OS of 30.4 months.

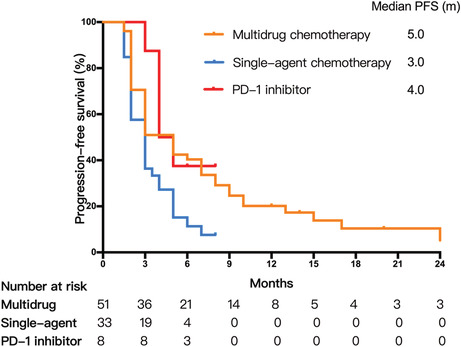

## 1 INTRODUCTION

Thymic carcinoma is an uncommon malignant tumor of thymic epithelial cells with a prevalence of 0.02 cases per 100,000 person‐years.^1^, ^2^ Thymic carcinoma has a higher degree of malignancy than thymoma and is more likely to produce distant metastasis. It was reported that the 5‐year survival rate of patients with thymic carcinoma was as low as 30%–50%.^3^ For advanced thymic carcinoma, chemotherapy remains the main treatment. Platinum‐based chemotherapy and anthracycline‐based chemotherapy have been proven to have high efficiency for thymic carcinomas as first‐line therapies, with a response rate between 20% and 50%.^4^, ^5^ Currently, based on the single‐arm phase II clinical trial, paclitaxel + platinum chemotherapy, the median survival time of which is 20.0 months, is the preferred option as the first‐line treatment.^6^, ^7^

There is no standard therapy recommended for thymic carcinoma patients who have failed first‐line platinum‐based chemotherapy. Previously reported studies have mainly tested single drugs with small sample sizes and limited efficacy. Single‐agent chemotherapies such as pemetrexed and S1 are the most reported salvage chemotherapy drugs, with a response rate of 19% to 30%.^8^, ^9^ Targeted treatments with drugs such as sunitinib and imatinib have shown limited activity.^10^, ^11^ Immune checkpoint inhibitors (ICIs) have been proven effective in thymic carcinoma, with a median progression‐free survival (PFS) of 3.8 to 6.1 months. However, grade 3 or higher immune‐related adverse events (ir‐AEs) were detected in 15% to 68% of patients.^12^, ^13^, ^14^ Multidrug chemotherapies were also applied to the second‐line thymic carcinoma treatment in real‐world practices and clinical trials.^15^, ^16^, ^17^, ^18^ The head‐to‐head comparisons are still lacking between different treatment options for metastatic thymic carcinoma.

The survival time of advanced thymic cancer patients receiving systematic treatments was long, with a median survival time of up to 49.4 months.^19^ More than half of the patients with thymic carcinoma underwent second‐line treatment after the failure of first‐line chemotherapy. Few studies focused on the second‐line therapy for thymic carcinoma. Only one study compared the different second‐line therapy options for thymic carcinoma.^17^ However, the patients in this study were from 1995 to 2014, and most patients received anthracycline‐based first‐line chemotherapy, which was not compatible with current clinical practice.^6^ Therefore, it is necessary to identify safe and effective therapeutics for the second‐line treatment.

This study is a retrospective multicenter analysis demonstrating the outcomes of different second‐line therapies. Comparisons were made between multidrug chemotherapy, single‐agent chemotherapy, and PD‐1 inhibitors in patients diagnosed with advanced thymic carcinoma failed in platinum‐based chemotherapy at the clinic.

## 2 MATERIALS AND METHODS

Patients who had been confirmed to have advanced thymic carcinoma progressed on the first‐line platinum‐based chemotherapy and received the second‐line therapy between January 2015 and May 2020 at the Peking University Cancer Hospital in Beijing, Guangdong Provincial People's Hospital and Sun Yat‐sen University Cancer Center in Guangzhou were included. Inclusion criteria included a histological diagnosis of advanced (Masaoka‐Koga stage IVa or IVb) thymic carcinoma^20^; age ≥ 18 years; confirmed disease progression after first‐line platinum‐based chemotherapy; received at least one cycle of systemic treatment as second‐line therapy; and evaluable clinical efficacy and survival information. Patients receiving anthracycline‐based first‐line chemotherapy were excluded.

This study received approval from the ethics committees of the Peking University Cancer Hospital in Beijing, Guangdong Provincial Peoples Hospital and Sun Yat‐sen University Cancer Center in Guangzhou.

PFS was calculated from the time from the beginning of the second‐line treatment to disease progression or death; OS was calculated from the beginning of the second‐line treatment to death or the latest follow‐up. Based on the Response Evaluation Criteria in Solid Tumors (RECIST) ver. 1.1, objective tumor response received computed tomography (CT) assessment every 6–8 weeks since the beginning of the treatment.

Kaplan–Meier methods were used to estimate the PFS and OS curves, and differences between different treatment groups were analyzed and compared by the log‐rank test. Pearson chi‐square or Fisher's exact chi‐square test was used to comparing the objective response rates between different treatment groups. Cox regression was used for the multivariate analyses in PFS and OS. Differences with a *p* < 0.05 were considered to be statistically significant. SPSS 24.0 software was used in all the statistical analyses and plots were created using PRISM 6 software.

## 3 RESULTS

### 3.1 Patient characteristics

Between January 2014 and May 2020, 115 patients received second‐line therapy from three thoracic cancer centers in China. The study excluded 21 patients who did not received first‐line platinum‐based chemotherapy. Another two patients were excluded for using an ineffective second‐line regimen (gefitinib). The analysis study included 92 patients in total. A summary of patient characteristics is illustrated in Table 1. The median age of the 92 patients was 54 (range: 18–77), with more men than women (60.9% vs. 39.1%, respectively). In terms of KPS score, the majority of patients scored 70–80 (53.3%). According to the classification of the Masaoka staging system, 28 (30.4%) patients were in stage IVA, and 64 (69.6%) were in stage IVB. In terms of distant metastases, lung metastasis was the most common (46.7%), followed by liver (30.4%) and bone (18.5%). All 92 patients underwent first‐line platinum‐based chemotherapy. The most frequent therapeutic regimen was gemcitabine + platinum (45/92, 48.9%), followed by paclitaxel + platinum (38/92, 41.3%) and pemetrexed + platinum (9/92, 9.6%).

**TABLE 1:** Patient characteristics

Variable	*N* = 92
Median age, years (range)	54 (18–77)
Sex	
Male	56 (60.9%)
Female	36 (39.1%)
KPS score	
90–100	35 (38.0%)
70–80	49 (53.3%)
≤60	7 (7.6%)
Disease stage (Masaoka‐Koga)	
IVa	28 (30.4%)
IVb	64 (69.6%)
Metastatic site	
Lung	43 (46.7%)
Lymph node	53 (57.6%)
Pleura	24 (26.1%)
Pericardial	11 (12.0%)
Bone	17 (18.5%)
Liver	27 (29.3%)
First‐line chemotherapy	
Gemcitabine + Platinum	45 (48.9%)
Paclitaxel + Platinum	38 (41.3%)
Pemetrexed + Platinum	9 (9.6%)

For second‐line therapy, multidrug chemotherapy was used in more than 50% of the patients (51/92, 55.4%). Thirty‐three patients (35.9%) received single‐agent chemotherapy. Eight patients (8.7%) underwent PD‐1 inhibitors as second‐line therapy, with six receiving pembrolizumab and two receiving nivolumab. The detailed regimens and cycles in the second‐line setting can be found in Table S1. Patients underwent a median of four cycles (range: 1–10) of second‐line treatment. Tumor progression was the main cause of treatment discontinuation (72/92, 85.9%). Fifty‐one of 94 patients (55.4%) received subsequent therapy after second‐line therapy failure. The most frequent subsequent therapy was S‐1 (12/51, 23.5%).

### 3.2 Response rates and survival outcomes of second‐line treatment

With a median follow‐up time of 26.7 months, the median PFS in the whole population was 3.0 months (95% CI, 2.2–3.8 months), and the median OS was 21.1 months (95% CI, 12.5–29.7 months) in those receiving second‐line therapy. We compared the response rates of different second‐line therapies in Table 2. The multidrug chemotherapy group performed better efficacy than the other two groups, with an ORR of 35.3% (*p* = 0.006). The ORRs was 6.0% in the single‐agent chemotherapy group and 12.5% in the PD‐1 inhibitor group. No CR was reached.

**TABLE 2:** The efficacies of second‐line treatments according to different regimes

Regimen	*N*	ORR	*p* value	Median PFS	*p* value	Median OS	*p* value
Multidrug chemotherapy	51	35.3%	0.006	5.0	0.008	30.4	0.045
Single‐agent chemotherapy	33	6.0%		3.0		16.4	
PD‐1 inhibitor	8	12.5%		4.0		Unreached	

**FIGURE 1:**
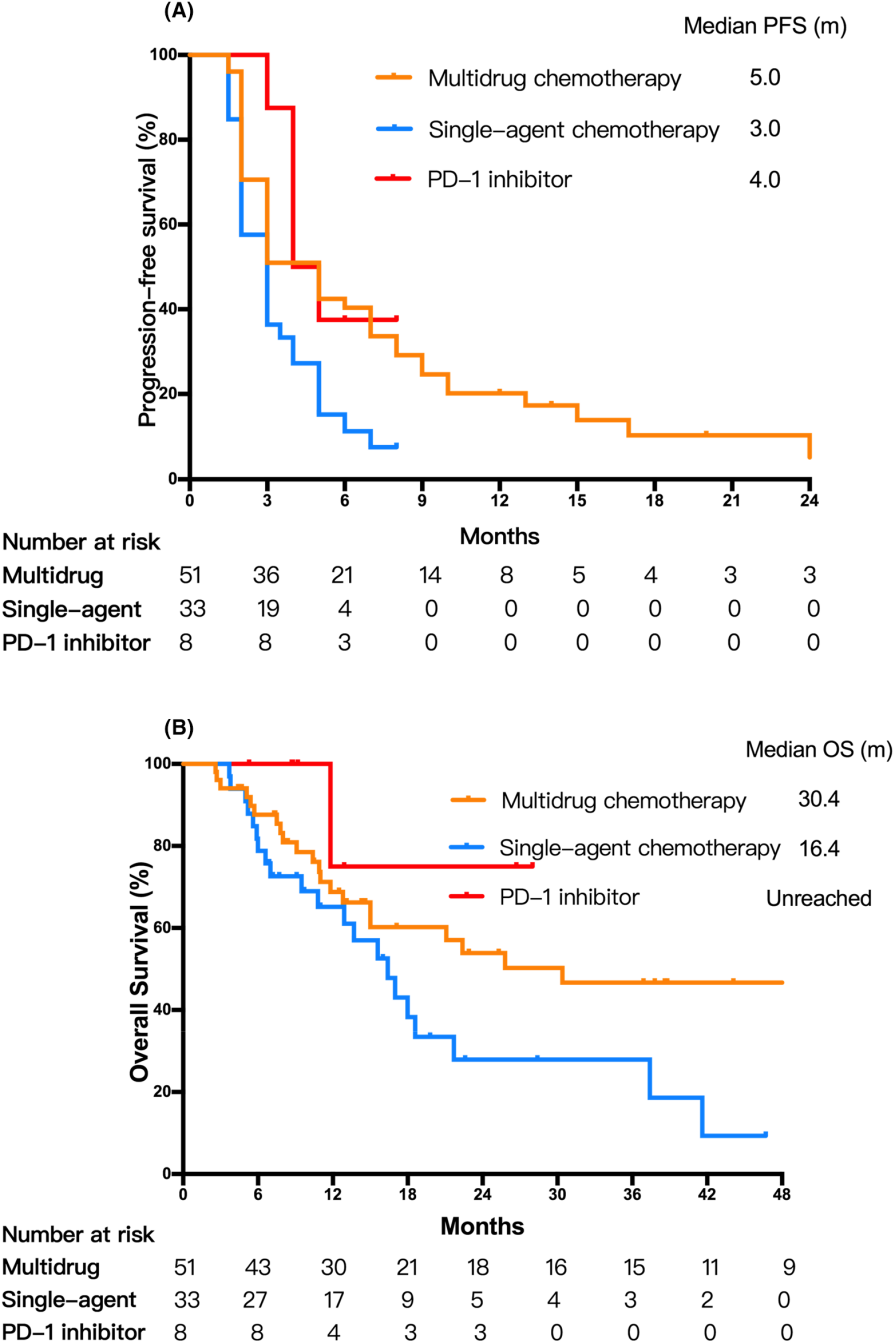
Kaplan–Meier curves for progression‐free survival (PFS) and overall survival (OS) in different treatment groups. (A) PFS in different treatment groups. (B) OS in different treatment groups.

The PFS and OS for different therapy groups are shown in Table 2 and Figure 1. The median PFS in the multidrug chemotherapy group was 5.0 months (95% CI, 3.5–6.5 months), showing a significant advantage compared to the other two treatment groups (*p* = 0.008). The median PFS in the single‐agent chemotherapy group and PD‐1 inhibitor group was 3.0 months (95% CI, 2.2–3.8 months) and 4.0 months (95% CI, 2.6–5.3 months), respectively. The patients in the multidrug chemotherapy group also showed an advantage in OS in comparison with the other two treatment groups (*p* = 0.045), with a median OS of 30.4 months (95% CI, 0–66.8 months). The median OS in the single‐agent chemotherapy group was 16.4 months (95% CI, 11.9–20.9 months). The median OS in the PD‐1 inhibitor group was not reached.

In the multidrug chemotherapy group, the majority of patients received docetaxel + platinum (41/51, 80.3%). Subgroup analysis indicated that docetaxel + platinum had a similar ORR compared to the other multidrug chemotherapies (36.6% vs. 30.0%, *p* = 0.696). Docetaxel + platinum had a trend of better PFS (*p* = 0.197) and OS (*p* = 0.203) than the other multidrug chemotherapies but failed to achieve statistical significance. In the single‐agent chemotherapy group, docetaxel was the most common drug (17/36, 47.2%). No difference was observed between docetaxel and other single‐agent chemotherapies in PFS (*p* = 0.141) and OS (*p* = 0.448).

To further investigate prognostic factors in patients with thymic carcinoma treated with second‐line treatment, we performed multivariate Cox regression analysis of PFS and OS, respectively. Factors such as age, sex, ECOG status, disease stage (Masaoka‐Koga) and second‐line treatment were included in the multivariate analysis of PFS, which confirmed that second‐line treatment (*p* = 0.035) was independent factor for PFS (Table 3). For OS, we included in the multivariate analysis whether or not treated with third‐line therapy. The result showed that KPS score (*p* = 0.000), disease stage (Masaoka‐Koga) (*p* = 0.014) and second‐line treatment (*p* = 0.037) were independent factors for OS (Table 4).

**TABLE 3:** Multivariate analysis of predictors on PFS in patients with thymic carcinoma receiving second‐line therapy

	*N* (%)	HR	95% CI	*p*
Age		1.002	0.98–1.025	0.856
Sex				
Male	56 (60.9%)			
Female	36 (39.1%)	0.947	0.586–1.53	0.824
KPS score				
90–100	35 (38.0%)			0.558
70–80	49 (53.3%)	0.643	0.274–1.468	0.287
≤60	7 (7.6%)	0.567	0.24–1.337	0.195
Disease stage (Masaoka‐Koga)				
IVa	28 (30.4%)			
IVb	64 (69.6%)	1.377	0.81–2.34	0.237
Second‐line treatment				
Multidrug chemotherapy	51 (55.4%)			0.035
Single‐agent chemotherapy	33 (35.9%)	1.305	0.505–3.369	0.583
PD‐1 inhibitor	8 (8.7%)	2.391	0.914–6.257	0.076

**TABLE 4:** Multivariate analysis of predictors on OS in patients with thymic carcinoma receiving second‐line therapy

	*N* (%)	HR	95% CI	*p*
Age		0.998	0.969–1.028	0.917
Sex				
Male	56 (60.9%)			
Female	36 (39.1%)	1.762	0.910–3.413	0.175
KPS score				
90–100	35 (38.0%)			0.000
70–80	49 (53.3%)	1.323	0.66–2.65	0.43
≤60	7 (7.6%)	8.232	2.908–23.3	0.000
Disease stage (Masaoka‐Koga)				
IVa	28 (30.4%)			
IVb	64 (69.6%)	2.581	1.211–5.501	0.014
Second‐line treatment				
Multidrug chemotherapy	51 (55.4%)			0.037
Single‐agent chemotherapy	33 (35.9%)	2.808	0.37–21.328	0.318
PD‐1 inhibitor	8 (8.7%)	5.875	0.763–45.209	0.089
Receiving third‐line therapy				
Yes	51 (55.4%)			
No	41 (44.6%)	0.753	0.398–1.426	0.385

### 3.3 Adverse events due to second‐line treatment

**FIGURE 2:**
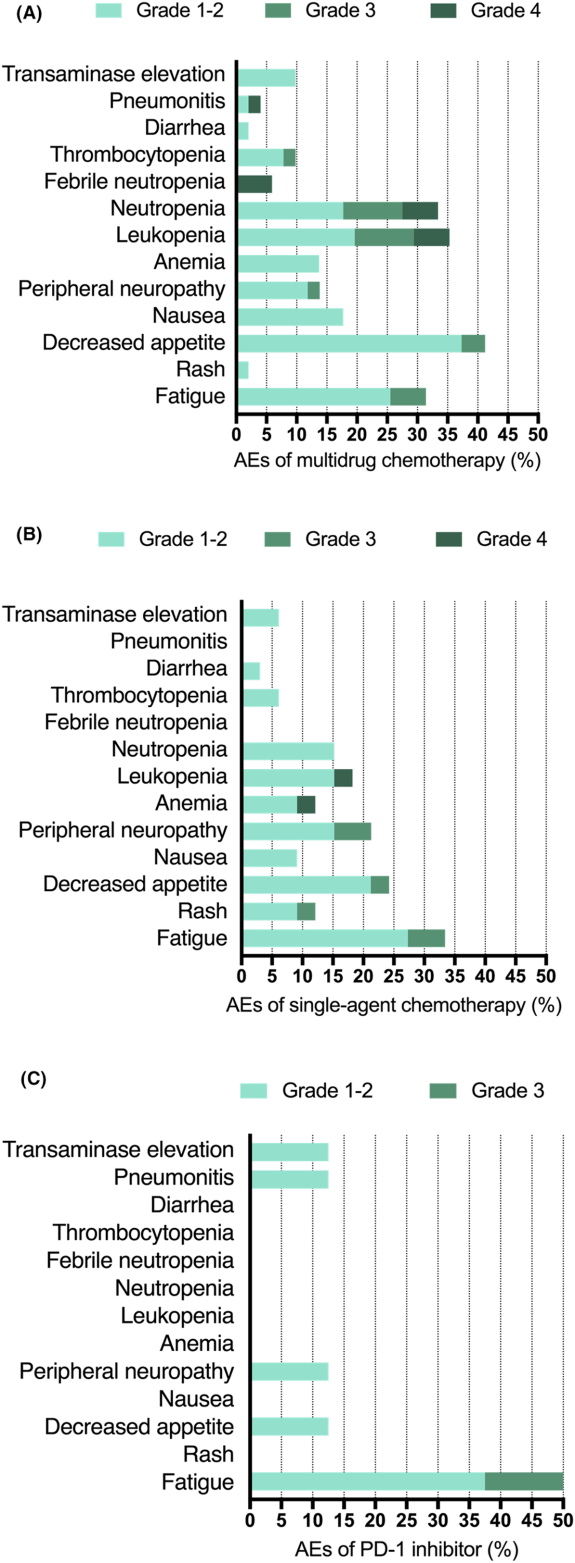
Treatment‐related adverse events (AEs). (A) AEs in the multidrug chemotherapy group. (B) AEs in the single‐agent chemotherapy group. (C) AEs in the PD‐1 inhibitor group.

Adverse events (AEs) in the different therapy groups are summarized in Figure 2. The most prevalent adverse events related to treatment in the multidrug chemotherapy group were decreased appetite (41.2%), fatigue (31.4%), leukopenia (35.3%), neutropenia (33.4%), nausea (17.7%), peripheral neuropathy (13.8%), and anemia (13.7%). Adverse effects in the single‐agent chemotherapy group were mainly fatigue (33.4%), decreased appetite (24.2%), peripheral neuropathy (21.3%), leukopenia (18.2%), and neutropenia (18.2%). Fatigue (50%) was the most common adverse event among the eight patients receiving PD‐1 inhibitors. In addition, one patient developed immune‐related pneumonitis, and one patient developed immune‐related hepatitis. Grade 3–4 AEs showed the highest occurrence in patients receiving platinum‐based chemotherapy and were primarily hematologic. Neither of the groups reported treatment‐related death.

## 4 DISCUSSION

Due to the rarity of thymic carcinoma and the low feasibility of large‐scale clinical trial, there is no standard treatment for second‐line therapy in thymic carcinoma patients. In fact, thymic carcinoma patients had a relatively long survival time after the first‐line treatment failed, with a reported median OS of second‐line treatment between 14.5 months and 27.4 months.^8^, ^12^, ^17^, ^21^ Most patients with advanced thymic carcinoma can receive second‐line treatment. Previous clinical researches on the second‐line treatment of thymic tumors showed insufficient efficacy in thymic carcinoma and lacked head‐to‐head comparisons.^8^, ^9^, ^10^, ^22^ Therefore, the identification of better treatment options for second‐line treatment is important.

Our study first focused on second‐line therapy in thymic carcinoma after the failure of platinum‐based chemotherapy. Compared to single‐agent chemotherapy and PD‐1 inhibitors, multidrug chemotherapy had a high PFS (5.0 months, 95% CI, 3.5–6.5; *p* = 0.008) and OS (30.4 months, 95% CI, 0–66.8; *p* = 0.045). Compared with the other therapy groups, the docetaxel + platinum group presented a significantly better ORR of 35.3% (*p* = 0.006). We performed multivariate survival analyses and showed that patients benefit from both PFS and OS when treated with multidrug chemotherapy as a second‐line treatment. Second‐line therapy was the only factor that was shown to have a statistically significant impact on PFS in a multivariable analysis. However, for OS, KPS score, stage and second‐line therapy were all independent prognostic factors. We also included the third‐line therapy in our multivariate analysis of OS, and regrettably, with or without triple therapy, patients showed no statistically significant difference in OS. Thymic carcinoma has a longer survival period than non‐small cell lung cancer (NSCLC) after failing platinum‐based treatment. Thus, combined therapy may be considered for second‐line therapy in thymic carcinoma patients, and single‐agent treatment may be administered as third‐line or later treatment. A recent multicenter study^17^ had a similar result: as opposed to monotherapy, platinum doublet therapy had a high response rate in second‐line treatment of thymic carcinoma. But no survival benefit were observed in that study. The main reasons may be the long time span (1995–2014) and the diversity of first‐line treatment.

In our study, docetaxel was the most common chemotherapy drug, in both multidrug group and single agent group. However, subgroup analysis did not find the discrepancies in PFS and OS compared to other regimens when patients were treated with docetaxel‐containing treatment. Docetaxel is widely accepted and applied as second‐line chemotherapy in NSCLC patients. A small case study (*n* = 15) reported a response rate of 26.7% for docetaxel alone or in combination with platinum as a second‐line treatment for advanced thymic carcinoma.^23^ However, docetaxel did not show an advantage over other chemotherapy drugs in the second‐line treatment of thymic carcinoma.

Our study included eight patients who underwent PD‐1 inhibitors as second‐line treatment. Giaccone et al. reported a phase II trial in which patients with advanced thymic carcinoma were treated with pembrolizumab.^24^ Survival analysis showed that the median PFS was 4.2 months, whereas the one‐year OS turned out as 71%, which is consistent with our results (median PFS = 4 months). Due to the relatively short application time of immune checkpoint inhibitors in thymic carcinoma, the OS of the PD‐1 inhibitor group was not reached. PD‐1 inhibitors are a promising treatment option for thymic carcinoma patients that had not gained successful outcomes in the platinum‐based treatment. It should be noted, however, that severe immune‐related events occurred more frequently in patients with thymic carcinoma than patients with other types of tumor. Approximately 15% of patients with thymic carcinoma suffer critical autoimmune toxicities when receiving PD‐1 inhibitor therapy, including 5%–9.1% with myocarditis.^12^, ^24^ In our study, no myocarditis was observed, but one patient (12.5%) developed immune‐related pneumonitis, and one patient (12.5%) had transaminase elevation.

In our study, multidrug chemotherapy was well tolerated, and grade 3–4 adverse events did not show notable differences from those reported in other second‐line treatments. Grade 3–4 toxicities were hematologic and reversible. There were some limitations in our study. The retrospective design is the major limitation. However, thymic carcinoma is a less common tumor, and it is considerably more challenging to organize large sample prospective studies, especially for second‐line therapy. The accurate and rigorous selection of patients might reduce the inherent biases of this retrospective study.

## 5 CONCLUSIONS

Multidrug chemotherapy had a trend for better ORRs and more positive survival outcomes than single‐agent chemotherapy and PD‐1 inhibitors. If well tolerated, multidrug chemotherapy has great potential as the second‐line therapy in patients with thymic carcinoma. Further prospective studies are needed to determine the optimal platinum‐based chemotherapy regimen for second‐line treatment in thymic carcinoma.

## AUTHOR CONTRIBUTIONS

Yang Wang and Jian Fang planned and designed the study. Yang Wang Xuanye Zhang and Dan Tian conducted the data collection and analysis and wrote the article. Jun Nie, Ling Dai, Weiheng Hu, Xiaoling Chen, Xiangjuan Ma, and Guangming Tian collected data. Sen Han, Di Wu helped to conduct the literature review. Ziran Zhang, Jie Zhang assisted in data analysis. All authors read and approved the final manuscript of the study.

## CONFLICT OF INTEREST

The author has no conflicting interests to disclose with the content of this article.

## ETHICS STATEMENT

This study was approved by institutional ethics committees of participant centers. Because of the retrospective character of this study, there was no requirement for informed consent.

## Supporting information


Table S1

## Data Availability

The analytic dataset for this study is available from the authors upon reasonable request. Due to ethics‐related regulations, the data may contain patient privacy and are therefore not available to the public.
