# Supplementary material for: Second‐line treatment options in advanced thymic carcinoma after failure of platinum‐based chemotherapy: A multicenter retrospective study
Source: Cancer Med. 2022 Aug 4;12(3):2303–11. doi: 10.1002/cam4.5053 (PMC9939196; doi:10.1002/cam4.5053)
Supplement: Supplementary file 1 — Table S1 [file CAM4-12-2303-s001.docx]

Supplemental Table 1. Second-line Chemotherapy for Thymic Carcinoma

| Regimen | Dosage and Interval | Median cycles  （Range） | | N=92 |
| --- | --- | --- | --- | --- |
| Docetaxel + Platinum | Docetaxel 75mg/m2, d1; | | 4(2-8) | 41(44.6%) |
| Docetaxel | 75mg/m2, every 21 days | | 4(2-10) | 17(18.5%) |
| S1 | < l.25 m2,80 mg/day;  ≥ 1.25 to <1.5 m2, 100mg/day;  ≥ 1.5 m2, 120mg/day | | 5(2-8) | 7(7.6%) |
| Pembrolizumab | 200mg, every 21 days | | 5(4-6) | 4(4.3%) |
| Nivolumab | 3mg/kg, every 14 days | | 7(6-8) | 2(2.2%) |
| Gemcitabine | 1250mg/m2, d1, d8, every 21 days | | 4(2-6) | 9(9.8%) |
| Gemcitabine + Cisplatin | Gemcitabine 1250mg/m2, d1, d8; Cisplatin 75mg/m2, d1; every 21 days | | 3(1-5) | 4(4.3%) |
| Paclitaxel + Carboplatin | Paclitaxel 175mg/m2, Carboplatin AUC = 4-6; every 21 days | | 3(2-3) | 4(4.3%) |
| Cisplatin + Cyclophosphamide + Doxorubicin | Cisplatin 50 mg/m2, Doxorubicin 50 mg/m2, Cyclophosphamide 500 mg/m2, every 21 days | | 3(1-4) | 2(2.2%) |
